# Supplementary material for: Resensitization of Fosfomycin-Resistant Escherichia coli Using the CRISPR System
Source: Int J Mol Sci. 2022 Aug 16;23(16):9175. doi: 10.3390/ijms23169175 (PMC9409345; doi:10.3390/ijms23169175)
Supplement: Supplementary file 1 [file ijms-23-09175-s001.zip › ijms-1848244-supplementary.pdf]

SUPPLEMENTARY MATERIAL

Article

Resensitization of fosfomycin-resistant *Escherichia coli* using the CRISPR system

Haniel Siqueira Mortagua Walflor [1,3], Aline Rodrigues Castro Lucena[1], Felipe Francisco Tuon[2], Lia Carolina Soares Medeiros[3], and Helisson Faoro [1,4*]

[1]Laboratory for Applied Science and Technology in Health, Carlos Chagas Institute, Fiocruz, Curitiba, PR, 81350-010, Brazil;
[2]Laboratory of Emerging Infectious Diseases, Pontifícia Universidade Católica do Paraná, Curitiba, PR, Brazil;
[3]Laboratory of Cell Biology, Carlos Chagas Institute, Fiocruz, Curitiba, PR, 81350-010, Brazil;
[4]Graduate Program on Bioinformatics, Federal University of Paraná, Curitiba, PR, 81520-260, Brazil.
* Correspondence: helisson.faoro@fiocruz.br.

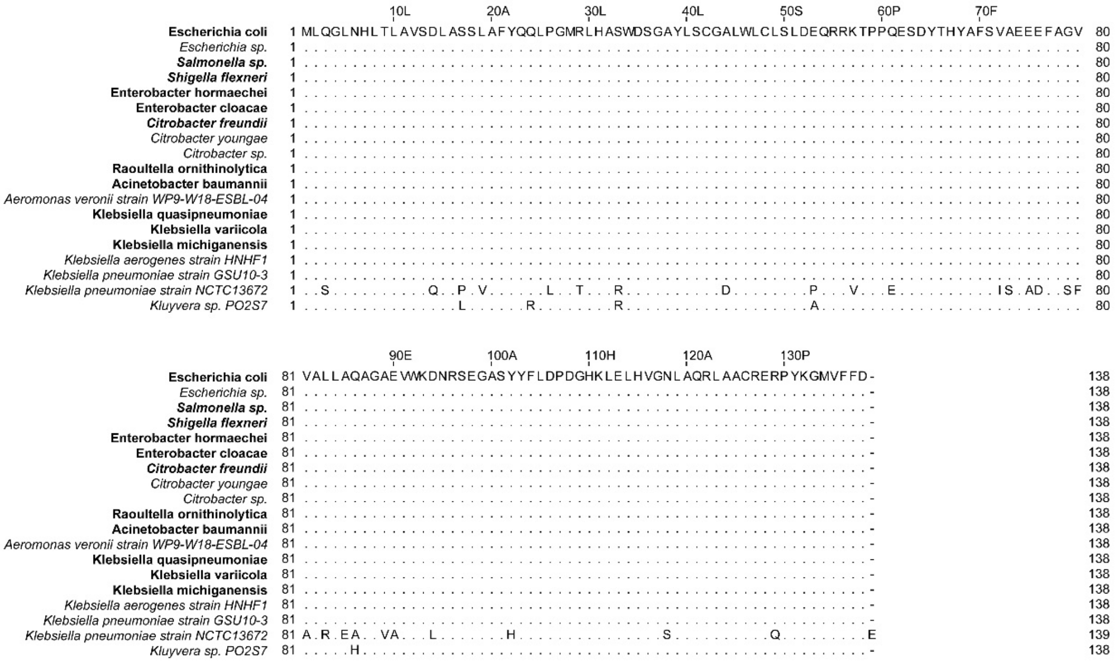

Figure S1. Alignment of FosA3 protein sequences between different enterobacteria isolates. The dotted region represents conserved amino acids.

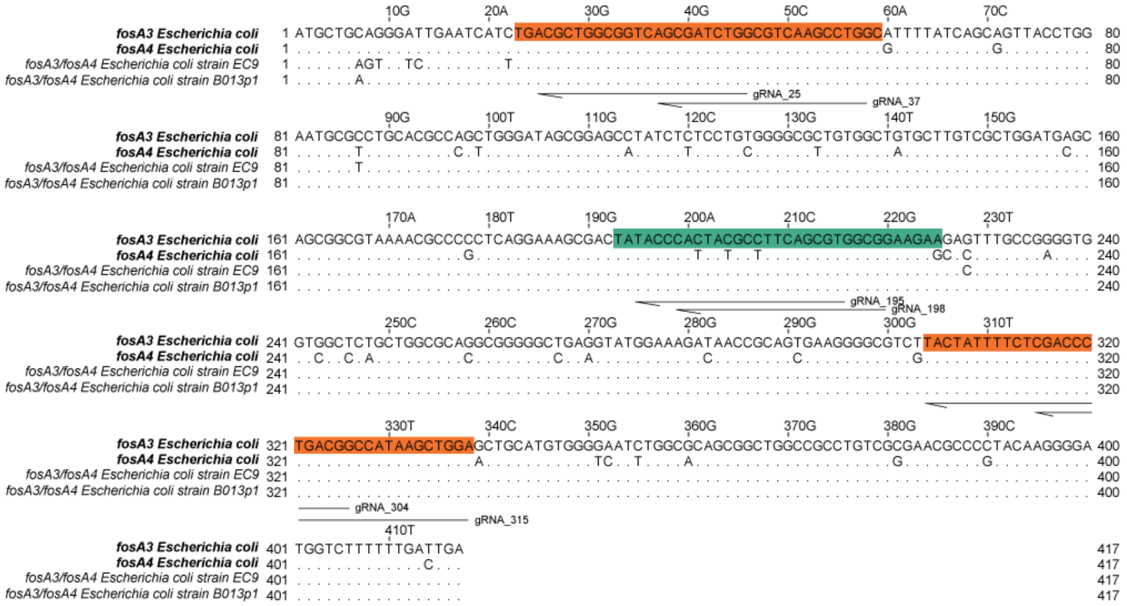

Figure S2. Annealing region of the gRNAs targeting the *fosA3* allele used in this work. In green: 100% conserved protospacer region in *fosA3* alleles in which the most efficient gRNAs anneal. In orange: 100% conserved protospacer region between the *fosA3* and *fosA4* sequences in which two gRNAs anneal. The arrows represent the spacer region of the gRNAs, while the orientation of the arrow to the left indicates annealing on the positive strand of the target DNA.

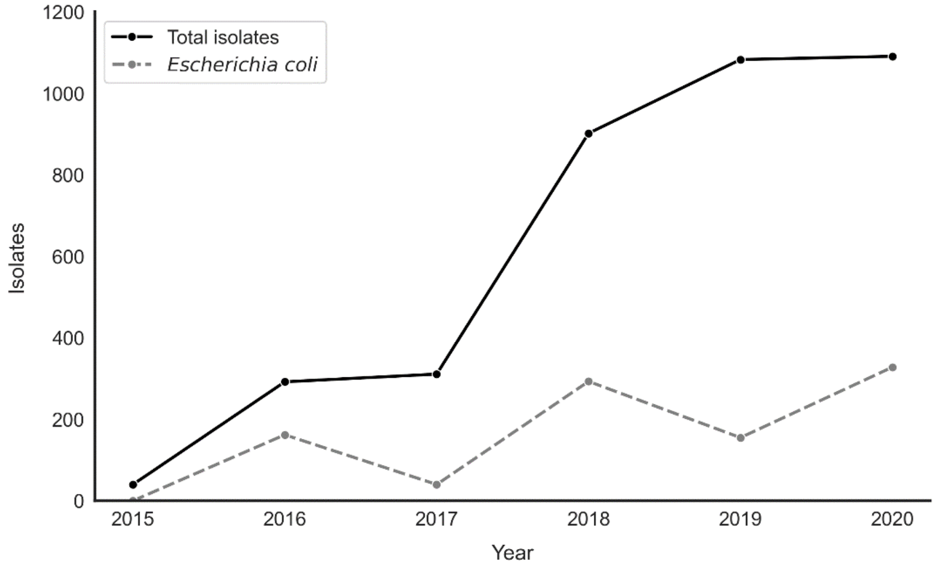

Figure S3. Incidence of *fosA3*+ isolates between 2015 and 2020.

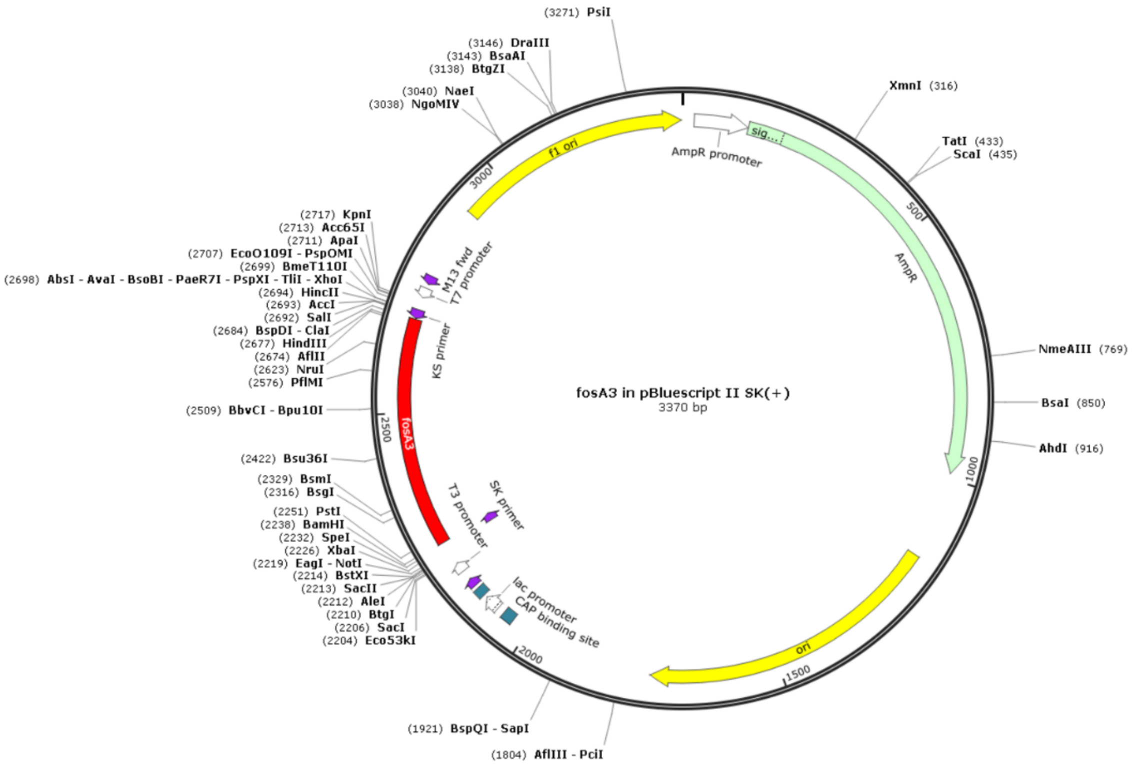

Figure S4. pFOSA3 vector map. Ori/F1 ori (yellow): vector replication origins; *ampR* (green): ampicillin resistance gene; *fosA3* (red): fosfomycin resistance gene.

```
                                             10L       20A       30L       40L       50S
FosA3_[bacteria]                    1 MLQGLNHLTLAVSDLASSLAFYQQLPGMRLHASWDSGAYLSCGALWLCLSLDEQRRKTPP 60
FosA4_[Enterobacteriaceae]          1 .......................R........R...................A......A 60
FosA_[Enterobacterales]             1 ...S...........QK.VT.WHE.L.LT...R.NT....T..D..V...Y..A.QYV.. 60
FosA2_[Enterobacterales]            1 ...S...........QK.VT.WHE.L.LT...R.NT....T..D..V...Y..A.GYV.. 60
FosA8_[Escherichia_coli]            1 ..NA.........N.PA.IT.WRD.L.L....E.HT....T..D......Y..T.TFI.. 60
FosA5_[Enterobacteriaceae]          1 ..S..........Q..P.V......L..M...R..........D........P...V... 60
FosA_[Enterobacteriaceae]           1 ..S..........Q..P.V......L..T...R..........D........P...V... 60
FosA6_[Escherichia_coli]            1 ..S..........Q..P.V......L..T...R..........D........P...V... 60
FosA_[Enterobacteriaceae]           1 ..S..........Q..P.V......L..T...R..........D........P...V... 60
FosA_[Klebsiella]                   1 ..S..........Q..P.V......L..T...R..........D........P...V... 60
FosA_[Raoultella]                   1 ..S..........S..P.V...H..L..T...R..G.......D........P...V... 60
FosA_[Pseudomonas]                  1 ..T.........A..PA.I...RD.L.F..E.R..Q....EL.S......REP.---YGG 57
FosA_[Cronobacter_turicensis]       1 ..S.........R.V.R......S...L....R..G.......S.........N---AGS 57
FosA7_[Salmonella]                  1 ...S.........N.QT..T.WRD.L.LQ...E..T....T..D..V...Y.VSCNYVA. 60
FosA7.7_[Salmonella_enterica]       1 ...S.........K.QT..T.WRD.L.LQ...E..T....T..D..V...Y.VSCNYVA. 60
FosA7.2_[Salmonella]                1 ...S.........N.QT..T.WRD.L.LQ...E..T....T..D..V...Y.VS.NYVA. 60
FosA7.6_[Salmonella_enterica]       1 ...S.........N.QT..T.WRD.L.LQ...E..T....T..D..V...Y.VS.NYVA. 60
FosA7.8_[Salmonella_enterica]       1 ...S.........N.QT..T.WRD.L.LQ...E..T....T..D..V...Y.VS.NYVA. 60
FosA7.4_[Salmonella_enterica]       1 ...S.........N.QT..T.WRD.L.LQ...E..T....T..D..V...Y.VS.NYVA. 60
FosA7.3_[Salmonella]                1 ...S.........N.QT..T.WRD.L.LQ..VE..T....T..D..V...Y.VS.NYVA. 60
FosA7.5_[Enterobacteriaceae]        1 ...S.........N.Q...T.WRD.L.LQ...E.GT....T..D......Y.VS.SYVA. 60

                                             70F       80V       90E       100A      110H
FosA3_[bacteria]                   61 QESDYTHYAFSVAEEEFAGVVALLAQAGAEVWKDNRSEGASYYFLDPDGHKLELHVGNLA 120
FosA4_[Enterobacteriaceae]         61 ...............H..E...Q..H...............................H.. 120
FosA_[Enterobacterales]            61 ..........T....D.EPLSQR.E...VTI..Q.K.....F...............S.. 120
FosA2_[Enterobacterales]           61 ..........T..A.D.EPFSHK.E...VT...Q.K.....F...............S.. 120
FosA8_[Escherichia_coli]           61 .N..........EP.H.DA.AQK.KD..VT...E.K.....F...............D.. 120
FosA5_[Enterobacteriaceae]         61 E..........IS.AD..SFA.R.EA..VA...L.......H...............S.. 120
FosA_[Enterobacteriaceae]          61 E..........IS.AD..SFA.R.EA..VAI..L.......H...............S.. 120
FosA6_[Escherichia_coli]           61 E..........IS.AD..SFA.R.EA..VAI..L.......H...............S.. 120
FosA_[Enterobacteriaceae]          61 E..........IS.AD..SFA.R.EA..VAI..L.......H...............S.. 120
FosA_[Klebsiella]                  61 D..........IS.AD..SFAVR.EA..VAM..L.......H...............S.. 120
FosA_[Raoultella]                  61 E.............AD...FS.R.ET..VA...V.......................S.. 120
FosA_[Pseudomonas]                 58 PAA.......GI.AAD..RFA.Q.RAH.VRE..Q.....D.F........R..A...D.R 117
FosA_[Cronobacter_turicensis]      58 PAA.......T.S.AD.PQ..ET.TA..VTP..T.....D.W........Q......S.. 117
FosA7_[Salmonella]                 61 ..C........I.P.D.EPFSYK.K...VT.....K...Q.F...............D.. 120
FosA7.7_[Salmonella_enterica]      61 ..C........I.P.D.EPFSYK.K...VT.....K...Q.F...............D.. 120
FosA7.2_[Salmonella]               61 ...........I.P.D.EPFSCK.K...VT.....K...Q.F...............D.. 120
FosA7.6_[Salmonella_enterica]      61 ...........I.P.D.EPFSCK.K...VT.....K...Q.F...............D.. 120
FosA7.8_[Salmonella_enterica]      61 ...........I.P.D.EPFSCK.K...VT.....K...Q.F...............D.. 120
FosA7.4_[Salmonella_enterica]      61 ...........I.P.D.EPFSCK.K...VT.....K...Q.F...............D.. 120
FosA7.3_[Salmonella]               61 ...........I.P.D.EPFSYK.K...VT.....K...Q.F...............D.. 120
FosA7.5_[Enterobacteriaceae]       61 .K.........I.P.D.EPFSYK.K.S.VT.....K...Q.F...............D.. 120

                                             130P      +2
FosA3_[bacteria]                  121 QRLAACRERPYKGMVFFD--- 138
FosA4_[Enterobacteriaceae]        121 ..................--- 138
FosA_[Enterobacterales]           121 A.......K..A....TSDEA 141
FosA2_[Enterobacterales]          121 A.......K..A....TSDEA 141
FosA8_[Escherichia_coli]          121 A.......K..A....TSDEA 141
FosA5_[Enterobacteriaceae]        121 ........Q........AE-- 139
FosA_[Enterobacteriaceae]         121 ........Q........AE-- 139
FosA6_[Escherichia_coli]          121 ........QQ........Q-- 139
FosA_[Enterobacteriaceae]         121 ........Q.........Q-- 139
FosA_[Klebsiella]                 121 ........Q.........Q-- 139
FosA_[Raoultella]                 121 L.......Q.......Y.E-- 139
FosA_[Pseudomonas]                118 S......QA..A..R.A.--- 135
FosA_[Cronobacter_turicensis]     118 T......AA.....R.Y.--- 135
FosA7_[Salmonella]                121 S...Q...K..S..R.GPGK- 140
FosA7.7_[Salmonella_enterica]     121 S...Q...K..S..R.GPGK- 140
FosA7.2_[Salmonella]              121 S..TQ...K..S..R.GPGK- 140
FosA7.6_[Salmonella_enterica]     121 S..TQ......S..R.GPGK- 140
FosA7.8_[Salmonella_enterica]     121 S..TQ......S..R.GLGK- 140
FosA7.4_[Salmonella_enterica]     121 S...Q...K..S..R.GPGK- 140
FosA7.3_[Salmonella]              121 S..TQ......S..R.GPGK- 140
FosA7.5_[Enterobacteriaceae]      121 S...Q......S..R.GPGK- 140
```

Figure S5. Alignment of reference FosA protein sequences. The dotted region represents conserved amino acids.

Table S1. Information on the main gRNAs based on estimated overall efficiency

gRNA	Sequence (5′ - 3′)	Gene	Position	Strand	GC (%)	Ranking	Estimated efficiency
gRNA_285	AGTAAGACGCCCCTTCACTG	*fosA3*	285	-	55	1	72.17%
gRNA_195*	TACCCACTACGCCTTCAGCG	*fosA3*	195	+	60	2	71.15%
gRNA_198*	CCACTACGCCTTCAGCGTGG	*fosA3*	198	+	65	3	70.51%
gRNA_344	ATGTGGGGAATCTGGCGCAG	*fosA3*	344	+	60	4	68.55%
gRNA_108	CGGAGCCTATCTCTCCTGTG	*fosA3*	108	+	60	5	68.02%
gRNA_304	TACTATTTTCTCGACCCTGA	*fosA3/fosA4***	304	+	40	28	51.78%
gRNA_54	GTAACTGCTGATAAAATGCC	*fosA3/fosA4***	54	-	40	36	48.80%
gRNA_315	CGACCCTGACGGCCATAAGC	*fosA3/fosA4***	315	+	65	43	45.76%
gRNA_24	GACGCTGGCGGTCAGCGATC	*fosA3/fosA4***	24	+	70	52	39.31%

* gRNAs used in this work.
** gRNAs that anneal in 100% conserved regions in *fosA3*/*fosA4*.

Table S2. Proteins used to construct the phylogenetic tree and alignment.

Protein	ID
FosA (*Enterobactirales*)	WP_038415208.1
FosA2 (*Enterobactirales*)	WP_025205684.1
FosA3	WP_014839980.1
FosA4	WP_034169466.1
FosA8 (*Escherichia coli*)	WP_063277905.1
FosA5 (*Enterobacteriaceae*)	WP_012579083.1
FosA (*Enterobacteriaceae*)	WP_004214174.1
FosA6 (*Escherichia coli*)	WP_069174570.1
FosA (*Enterobacteriaceae*)	WP_004146118.1
FosA (*Klebsiella spp.*)	WP_009486251.1
FosA (*Raoultella*)	WP_004857515.1
FosA (*Pseudomonas spp.*)	WP_003082280.1
FosA (*Cronobacter turicensis*)	WP_015741374.1
FosA7 (*Salmonella spp.*)	WP_000941934.1
FosA7.1 (*Salmonella enterica*)	WP_058653118.1
FosA7.2 (*Salmonella spp.*)	WP_000941935.1
FosA7.6 (*Salmonella enterica*)	WP_061377147.1
FosA7.8 (*Salmonella enterica*)	WP_079820715.1
FosA7.4 (*Salmonella enterica*)	WP_023216493.1
FosA7.3 (*Salmonella spp.*)	WP_023231494.1
FosA7.5 (*Enterobacteriaceae*)	WP_000941933.1
